# Supplementary material for: Selection and Identification of an ssDNA Aptamer for Fibroblast Activation Protein
Source: Molecules. 2023 Feb 9;28(4):1682. doi: 10.3390/molecules28041682 (PMC9965484; doi:10.3390/molecules28041682)
Supplement: Supplementary file 1 [file molecules-28-01682-s001.zip › molecules-2096994-supplementary.pdf]

Supplemental Information

Selection and identification of an ssDNA aptamer for fibroblast activation protein

Xiaomin Zhang [1], Ge Yang [1, 2] *, Yi Zhao [1], Xuyan Dai [3], Wenjing Liu [4], Feng Qu [1], Yuanyu Huang [1]*

[1] School of Life Science; Advanced Research Institute of Multidisciplinary Science; Key Laboratory of Molecular Medicine and Biotherapy; Key Laboratory of Medical Molecule Science and Pharmaceutics Engineering; Beijing Institute of Technology, Beijing 100081, China.
[2] CAMS Key Laboratory of Antiviral Drug Research, Beijing Key Laboratory of Antimicrobial Agents, NHC Key Laboratory of Biotechnology of Antibiotics, Institute of Medicinal Biotechnology, Chinese Academy of Medical Sciences and Peking Union Medical College, Beijing 100050, China.
[3] Hunan Agricultural University, Changsha 410128, China.
[4] Beijing Key Laboratory of Drug Resistance Tuberculosis Research, Beijing Tuberculosis and Thoracic Tumor Research Institute, and Beijing Chest Hospital, Capital Medical University, Beijing 101125, China
* Correspondence: yyhuang@bit.edu.cn (Y. H.) or yangge@imb.cams.cn (G. Y.)

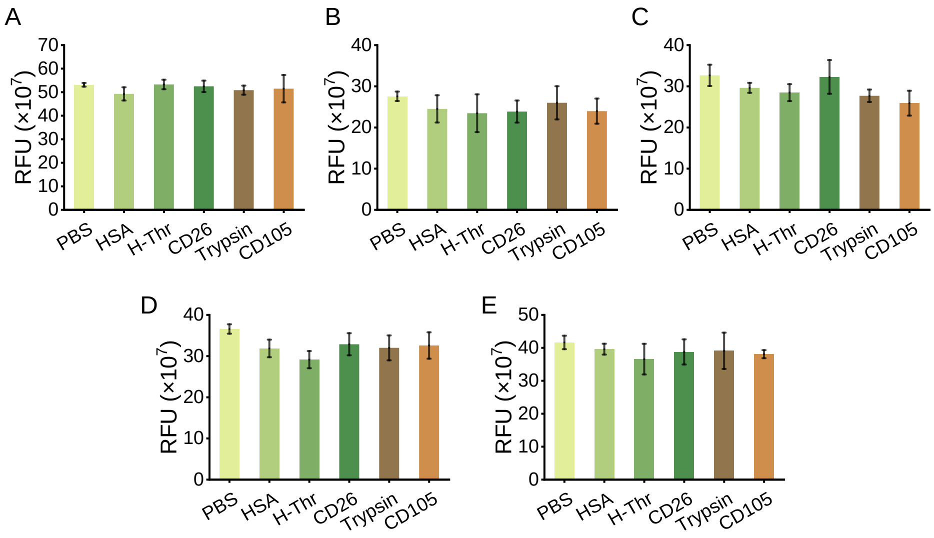

Figure S1. FAP-aptamers specificity evaluation. (A-E) show the evaluation of the interaction between AptFAP-A1~AptFAP-A5 and the five interfering proteins (HSA, H-Thr, CD26, Trypsin, CD105). FAM- fluorescentially labeled AptFAP-A1~AptFAP-A5 of 0.2 μmol/L was respectively mixed with five proteins of 2 μmol/L and incubated for 10 min at 37°C. The incubation mixture was analyzed by CE-LIF, where aptamers and PBS buffers (pH7.2) were incubated under the same conditions as control. Integrate the peak area of the free aptamer in each sample, and RFU is the unit of peak area.

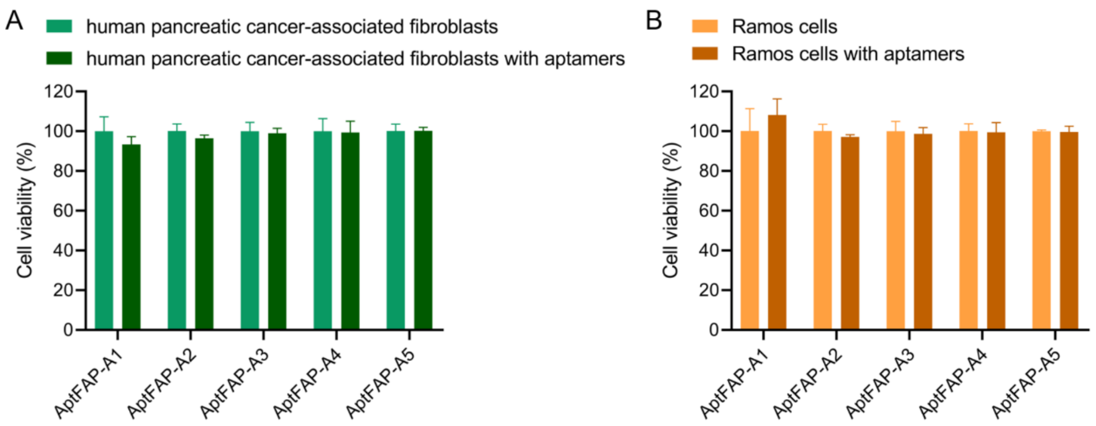

Figure S2. Effect of five candidate aptamers on cell growth. (A) Cell viability magnitude of 1 μmol/L candidate aptamers co-incubated with fibroblasts for 24 h; (B) Cell viability magnitude of 1 μmol/L candidate aptamers co-incubated with Ramos cells for 24 h.
